# Association of racism experience with gun purchase during COVID-19: Evidence from a national survey in the United States

**DOI:** 10.1016/j.pmedr.2024.102926

**Published:** 2024-11-09

**Authors:** Dejun Su, Khalid Alshehri, Brooke Lawson

**Affiliations:** a Department of Health Promotion, College of Public Health, University of Nebraska Medical Center, NE 68198, USA; b College of Public Health and Health Informatics, King Saud bin Abdulaziz University for Health Sciences, King Abdullah International Medical Research Center, Riyadh, Saudi Arabia; c Center for Reducing Health Disparities, College of Public Health, University of Nebraska Medical Center, NE 68198, USA

**Keywords:** Gun Purchase, Racism Experience, COVID-19, Firearm Violence, United States

## Abstract

**Objective:**

Concurrent with a substantial surge in gun purchases among Americans during COVID-19, there was an escalation of racism and hate crimes in the United States. Despite this disturbing trend, little is known about whether and the extent to which racism experience is linked to gun purchase during the pandemic. This study aims to examine the association between experience of racial discrimination and gun purchase among Americans during COVID-19.

**Methods:**

Based on data from the Health, Ethnicity, and Pandemic Survey (n = 2,584), a national survey conducted in the U.S. in October 2020, chi-square tests and logistic regressions were estimated to examine the association between racism experience and gun purchase during COVID-19 with and without controlling for selected covariates.

**Results:**

About 6.9 % of the sample reported gun purchases during COVID-19. Among respondents who reported experience of racism, 18.3 % purchased a gun as compared to 5.8 % among those who did not report experience of racism. Relative to respondents with no experience of racism, the odds of gun purchase for those who reported racism experience became 257 % as much (AOR = 2.57, 95 % CI: 1.63, 4.04) after controlling for other covariates in the analysis. Non-Hispanic Blacks were more likely to report gun purchases than non-Hispanic Whites (AOR = 1.80, 95 % CI: 1.04, 3.10).

**Conclusions:**

Americans who reported experience of racism during COVID-19 were far more likely to purchase a gun than those otherwise. These findings elevate the need for addressing racism as an important risk factor of firearm violence.

## 1 Introduction

Cumulative evidence from a number of studies revealed a substantial increase in gun violence and associated injuries and deaths in the United States since the beginning of the COVID-19 pandemic.(Sun et al., 2022, Center, 2022, Ssentongo et al., 2021) Besides direct health threats, this historic pandemic also incurred fear and anxiety about economic uncertainty, social unrest, and personal safety in the public, which contributed to a tremendous surge in gun purchases among Americans. Background check analysis conducted by the Federal Bureau of Investigation (FBI) indicated a 42 % surge in gun purchases from March to June of 2020, as compared to the same period in 2019.(FBI. NICS Firearm Checks: Month/Year. https://www.fbi.gov/file-repository/nics_firearm_checks_-_month_year.pdf/view., 2020)Table 1.

**Table 1:** A comparison between US adults based on responses to gun purchase during COVID-19: The Health, Ethnicity, and Pandemic Survey, 2020 (N = 2,584).

Variables	All (N)	Gun purchase during COVID-19		
		Yes N (%)	No N (%)	p-value
	2,584	179 (6.91)	2,405 (93.09)	
Experience of racism during COVID-19				<0.01
No	2,359	137 (5.83)	2,222 (94.17)	
Yes	225	41 (18.22)	184 (81.78)	
Race/Ethnicity				0.27
Non-Hispanic White	1,590	103 (6.50)	1,487 (93.50)	
Hispanic	431	36 (8.40)	395 (91.60)	
Non-Hispanic Black	306	25 (8.08)	281 (91.92)	
Non-Hispanic Asian	163	6 (3.91)	157 (96.09)	
Other	94	8 (8.37)	86 (91.63)	
Age				<0.01
18–29	529	56 (10.51)	474 (89.49)	
30–49	858	93 (10.87)	765 (89.13)	
50–60	876	23 (2.57)	853 (97.43)	
70+	321	7 (2.20)	314 (97.80)	
Gender				<0.01
Male	1,264	122 (9.65)	1,142 (90.35)	
Female	1,320	57 (4.28)	1263 (95.72)	
Marital Status				0.36
Unmarried	1,272	93 (7.31)	1179 (92.69)	
Married	1,312	86 (6.52)	1226 (93.48)	
Education level				<0.01
Less than high school	258	24 (9.30)	234 (90.70)	
High school graduate or equivalent	705	63 (8.98)	642 (91.02)	
Vocational/tech school/some college	729	49 (6.67)	680 (93.33)	
Bachelor's degree or above	892	43 (4.78)	850 (95.22)	
Household Income				0.54
$24,999 or less	494	41 (8.27)	453 (91.73)	
$25,000-$59,999	784	51 (6.44)	733 (93.56)	
$60,000-$149,999	1061	73 (6.89)	988 (93.11)	
$150,000 or more	245	14 (5.77)	231(94.23)	
Country of birth				<0.01
In the US	2,309	175 (7.58)	2,133 (92.42)	
Outside the US	275	3 (1.24)	272 (98.76)	
Self-rated health				0.26
Excellent/Very Good/Good	2,232	159 (7.13)	2,073 (92.87)	
Fair/Poor	352	19 (5.50)	333 (94.50)	
Self-assessment of bodyweight changes during COVID-19				0.12
No change	965	62 (6.42)	904 (93.58)	
Gained weight	1,091	88 (8.05)	1,003 (91.95)	
Lost weight	528	29 (5.44)	499 (95.56)	
Political Party				<0.01
Democrat or Lean Democrat	1,662	75 (4.54)	1,587 (95.46)	
Independent	257	21 (8.05)	236 (91.95)	
Republican or Lean Republican	665	82 (12.39)	583 (87.61)	
Kessler Distress Scale				<0.01
No severe psychological distress	2,175	128 (5.88)	2,047 (94.12)	
Severe psychological distress	409	51 (12.41)	358 (87.59)	
General Political Ideology in the State (based on results from the 2020 Presidential Election)				<0.01
Red states	1,094	99 (9.04)	995 (90.96)	
Blue states	1,490	80 (5.34)	1,410 (94.66)	

Notes: p-values are based on Chi-square tests.

For many Americans, especially Americans of racial and ethnic minorities, the fight against COVID-19 coincided with a personal struggle to cope with increasing racism and hate crimes in the country. In 2020, a total of 5,227 race/ethnicity/ancestry-based hate crimes were reported to the FBI, a 32 % increase from 2019.(The United States Department of Justice., 2020) The corresponding increase in anti-Asian hate crimes during the same period was 77 %, followed by an increase of 49 % in anti-Black hate crimes. There was evidence that the experience of racial discrimination during COVID-19 was associated with gun purchase among Asian Americans.(Wu et al., 2022 Apr) However, little is known about whether the same association holds in the general US population. This study aims to assess the association between the experience of racial discrimination and gun purchase during COVID-19 among Americans based on nationally representative survey data.

## 2 Methods

### 2.1 Data

The primary source of data is from the “Health, Ethnicity, and Pandemic (HEAP) Survey,” a national survey conducted in the United States in October 2020 by the National Opinion Research Center (NORC) at the University of Chicago. The main purpose of the survey was to assess changes in physical and mental health, health behavior, healthcare access, and social determinants of health including exposure to racism during the pandemic, with oversampling of racial and ethnic minorities. The initial sample was randomly drawn from NORC’s AmeriSpeak Panel, a probability-based panel designed to be representative of the US non-institutionalized population.(National Opinion Research Center. How AmeriSpeak Households Are Sampled. https://amerispeak.norc.org/about-amerispeak/Pages/Panel-Design.aspx. Accessed March 2, 2023) A total of 2,709 respondents participated in the survey. Detailed information regarding the panel recruitment for the survey can be found in previous publications using HEAP data.(Chen et al., 2021, Shi et al., 2022, Zhang et al., 2021, Wen et al., 2023 May 25, Hill et al., 2021 Nov) The study was approved by the Institutional Review Board at the NORC.

The second source of data was the Electoral College results from the 2020 Presidential Election to denote the general political ideology in the state where the respondents live.(Archives, 2020) After merging data from the two sources and removing cases with missing values, the final sample consisted of 2,584 respondents with complete information on measured factors.

### 2.2 Measures

The outcome measure on gun purchase was based on the survey question: “Have you purchased a gun during the pandemic (yes or no)?” The main explanatory variable on the experience of racial discrimination was based on the survey question: “Have you personally experienced any discrimination or unfair treatment because of your racial or ethnic background during the COVID-19 pandemic (yes or no)?” These two measures, together with other measures in the HEAP survey, had been pilot-tested by both the study team and the data collection team at NORC before the implementation of the survey.

Other covariates at the individual level included demographics (age groups, gender, race/ethnicity, nativity, marital status), socioeconomic status (education level and household income), and health status (self-rated health, self-assessment of changes in body weight during the pandemic, and the K6 measure on psychological distress (Kessler et al., 2002 Aug). The political environment was denoted by whether a respondent living in a blue or red state based on results from the 2020 Presidential Election.

### 2.3 Statistical analysis

Descriptive analysis was performed to compare respondents who reported gun purchase during the pandemic and those otherwise in terms of the variables used in this study. P values based on chi-square tests were used to denote if the differences between the two groups were statistically significant. Multiple logistic regressions with odds ratios and their 95 % confidence intervals (CI) were estimated to examine the association between racism experience and gun purchase after controlling for other variables. The SAS 9.4 software was used to for data merging, cleaning, and analysis.(Institute and Inc, 2022) Poststratification weighting was applied to generate nationally representative estimates.

## 3 Results

Among the 2,584 respondents in the study sample, 179 or 6.91 % reported gun purchases during COVID-19. The bivariate analysis results revealed a significant association between perceived racism and gun purchase: among respondents who reported experience of racism, 18.25 % purchased a gun as compared to 5.83 % among those who did not report experience of racism. Relative to non-Hispanic White respondents, a higher proportion of Hispanic and non-Hispanic Black respondents reported gun purchases. There were significant associations between gun purchase and several other variables including age, gender, education, nativity, party affiliation, psychological distress, and the overall political ideology in the state.

The association between racism experience and gun purchase was further corroborated by results from logistic regressions, as indicated in Table 2. Based on results from the unadjusted model, relative to respondents with no racism experience, the odds of gun purchase for those who reported racism experience became 361 % as much (OR = 3.61, 95 % CI: 2.47, 5.27). The corresponding odds ratio became 257 % as much (AOR = 2.57, 95 % CI: 1.63, 4.04) in the adjusted model after controlling for the effect of other covariates. While incorporating other covariates into the analysis mitigated the association between racism experience and gun purchase, the association remained statistically significant in the adjusted model.

**Table 2:** Logistic regression results on the odds of gun purchase among US adults during COVID-19: The Health, Ethnicity, and Pandemic Survey, 2020 (N = 2,584).

Variables	**Model 1** (Unadjusted)			**Model 2** (Adjusted)*		
	Odds Ratio	95 % CI		Odds Ratio	95 % CI	
Experience of racism during COVID-19						
No	Reference			Reference		
Yes	3.61	2.47	5.27	2.57	1.63	4.04

*In the adjusted model (Model2), other variables included in the weighted logistic regression are age, race/ethnicity, gender, marital status, household income, country of birth, self-rated health, self-assessment of body weight changes during COVID-19, political party/affiliation, general political ideology in the state (based on results from the 2020 presidential election), and Kessler distress scale.

## 4 Discussion

The revealed association between racism experience and gun purchase among Americans during the early stage of COVID-19 is concerning. The differences in gun purchase are substantial between Americans who reported racism experience and those otherwise in that the former group were about three times more likely to purchase a gun. Some victims of racism might feel threatened and acquire firearms for reported personal protection. This also implies that the escalation of racism and hate crimes during COVID-19 could have contributed to the recent surge in gun purchases.(FBI. NICS Firearm Checks: Month/Year. https://www.fbi.gov/file-repository/nics_firearm_checks_-_month_year.pdf/view., 2020) Results from the current study revealed that Blacks were more likely to report gun purchases than Whites during the pandemic. A related finding from a previous study using the same HEAP survey data indicated that Blacks were far more likely to report experience of racism (19 %) than Whites (3 %).(Su et al., 2022 Aug 5).

Part of the association between racism experience and gun purchase can be mediated by psychological distress. Exposure to racism often leads to psychological distress including fear and anxiety,(Shi et al., 2022, Paradies et al., 2015 Sep 23, Pieterse et al., 2012 Jan) which could prompt motives for gun purchases. There is a need for supporting victims of racism and alleviating their distress, which might help dampen the association between racism experiences and gun purchase.

This study sheds some new light on the surge in gun violence during COVID-19. Long-term trend analysis revealed a positive correlation between gun ownership and firearm homicide rates in the United States.(Siegel M, Ross CS, King C 3rd. The relationship between gun ownership and firearm homicide rates in the United States, 2013) National estimates suggested that the first year of the pandemic witnessed a 34.3 % increase in firearm-related nonfatal injuries and a 28.4 % increase in firearm-related deaths.(Sun et al., 2022) The strong association between racism experience and gun purchase, as revealed in this study, underscores the relevance of addressing racism as a risk factor for gun violence. Among Americans who reported experience of racism during the pandemic, one of the commonly adopted coping strategies was to directly confront the perpetrators,(Siegel M, Ross CS, King C 3rd. The relationship between gun ownership and firearm homicide rates in the United States, 2013) which can lead to violence in severe cases.

## 5 Limitations

The data on gun purchase and other variables from the HEAP survey were based on self-report, so potential recall biases could impact the robustness of study findings. Moreover, the HEAP survey did not collect information on the reasons for gun purchase, which makes it impossible for us to qualitatively link racism experience with the motive for gun purchase. A related limitation is that the HEAP survey did not contain information on the exact timing of exposure to racism and gun purchase, further restricting our ability to infer causation. Finally, the measurement of racism experience in the HEAP survey was based on one single question. Although this measure has been used in several previous studies,(Shi et al., 2022, Zhang et al., 2021, Wen et al., 2023 May 25, Su et al., 2022 Aug 5) its reliability might not be as good as multi-item measures on racism experience.(Krieger et al., 2005 Oct) Future research can evaluate the robustness of our study findings using validated, multi-item measures on racism experience.

## 6 Conclusion

Americans who reported experience of racism during COVID-19 were far more likely to purchase a gun than those otherwise. This finding elevates the need for addressing racism as an important risk factor of firearm violence.

## CRediT authorship contribution statement

**Dejun Su:** Writing – review & editing, Writing – original draft, Supervision, Project administration, Methodology, Funding acquisition, Data curation, Conceptualization. **Khalid Alshehri:** Writing – review & editing, Writing – original draft, Software, Methodology, Formal analysis, Data curation. **Brooke Lawson:** Writing – review & editing, Writing – original draft, Conceptualization.

## Declaration of competing interest

The authors declare that they have no known competing financial interests or personal relationships that could have appeared to influence the work reported in this paper.

## Data Availability

Data will be made available on request.
